# Improved Antioxidant Capacity of Black Tea Waste Utilizing PlantCrystals

**DOI:** 10.3390/molecules26030592

**Published:** 2021-01-23

**Authors:** Abraham M. Abraham, Reem M. Alnemari, Jana Brüßler, Cornelia M. Keck

**Affiliations:** Department of Pharmaceutics and Biopharmaceutics, Philipps-Universität Marburg, Robert-Koch-Str. 4, 35037 Marburg, Germany; abraham.abraham@pharmazie.uni-marburg.de (A.M.A.); Alnemari@students.uni-marburg.de (R.M.A.); jana.bruessler@staff.uni-marburg.de (J.B.)

**Keywords:** plants, plants waste, *Camellia sinensis*, black tea, PlantCrystals, small-scale bead milling, nanonization, antioxidant capacity, ecofriendly pharmaceutics

## Abstract

Antioxidants are recommended to prevent and treat oxidative stress diseases. Plants are a balanced source of natural antioxidants, but the poor solubility of plant active molecules in aqueous media can be a problem for the formulation of pharmaceutical products. The potential of PlantCrystal technology is known to improve the extraction efficacy and antioxidant capacity (AOC) of different plants. However, it is not yet proved for plant waste. Black tea (BT) infusion is consumed worldwide and thus a huge amount of waste occurs as a result. Therefore, BT waste was recycled into PlantCrystals using small-scale bead milling. Their characteristics were compared with the bulk-materials and tea infusion, including particle size and antioxidant capacity (AOC) in-vitro. Waste PlantCrystals possessed a size of about 280 nm. Their AOC increased with decreasing size according to the DPPH (1,1-diphenyl-2-picrylhydrazyl) and ORAC (oxygen radical absorbance capacity) assays. The AOC of the waste increased about nine-fold upon nanonization, leading to a significantly higher AOC than the bulk-waste and showed no significant difference to the infusion and the used standard according to DPPH assay. Based on the results, it is confirmed that the PlantCrystal technology represents a natural, cost-effective plant-waste recycling method and presents an alternative source of antioxidant phenolic compounds.

## 1. Introduction

All human beings are exposed to oxidative stress during their lifespan, caused by byproducts of metabolism, environment and lifestyle-related factors [1]. It is characterized by the production of unstable and reactive oxygen species (ROS) that lead to cellular damage [1,2]. Thus, regulation of ROS generation can be a treatment option for oxidative stress disorders. Blocking the activated ROS-related signaling could be another option to prevent oxidative stress-related diseases. Antioxidants are a good treatment option and plants are considered a good and balanced source of natural antioxidants. However, many of their active constituents possess low aqueous solubility and thus formulating them in pharmaceutical or cosmetic products is problematic. 

**Figure 1:**
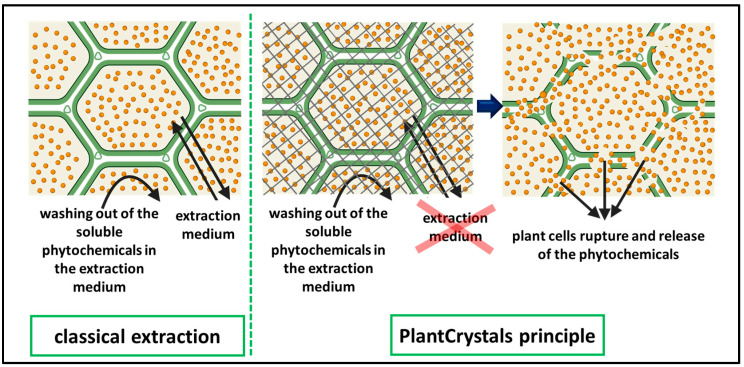
A scheme showing the principle of PlantCrystal technology in comparison with classical extraction process. The green lines represent the plant cells and the yellow round particles represent the phytochemicals inside the plant cells. The grey net (in the middle image) represents the forces applied during bead milling (BM) for plant cell rupture [8].

Recent studies showed that PlantCrystals, i.e., nanosized plant materials, are a novel formulation strategy to increase the solubility and potential of plant molecules and this has already been proved for many different plants in previous studies [3,4,5,6]. PlantCrystals are composed of plants, parts of plants or plant wastes and can be produced by different wet milling methods, i.e., bead milling or high-pressure homogenization [5]. The particle size of PlantCrystals is below 10 µm, which ensures the complete destruction of all intact plant cells leading to more efficient extraction of the plant material without the use of organic solvents (Figure 1) [5]. PlantCrystal technology is a promising, smart and easy method, which allows using the whole plants or even their wastes in pharmaceutics and cosmetics [3,4,5,7].

Using this ecofriendly method to improve the antioxidant capacity of plants is already proven [5]. The aim of this study was to go one step further and use plant waste to produce antioxidant formulations. Therefore, the waste of black tea (BT), *Camelia sinensis* (L.) from family *Theaceae*, was chosen.

Over the years, BT is used as an infusion of dried and fermented tea leaves in hot water and is considered the most consumed beverage worldwide after water [9,10]. Its extracts are also used to increase its therapeutic potential [11,12]. Black tea is usually used not only because of its taste, aroma and cultural practices but also due to its health values associated with its antioxidative properties. Tea is used for its antibacterial, hypocholesterolemic, anticarcinogenic and antiallergenic effects [13]. In addition, regular consumption of tea reduces inflammatory bowel, liver disorders, neurodegenerative diseases, diabetes, reduces the risk of cardiovascular disease and even triggers weight loss [13,14,15,16]. These effects are related to the rich phytochemicals in tea leaves, for example, polyphenols and flavonoids. 

Tons of tea-leaves waste are produced annually, because fresh prepared black tea infusion and/or ready-made tea (packed into cans and/or bottles) are consumed all over the world [17,18]. Most of the waste is burned, dumped into landfills or used as compost [19]. However, it may be considered a valuable source of antioxidant compounds. 

**Figure 2:**
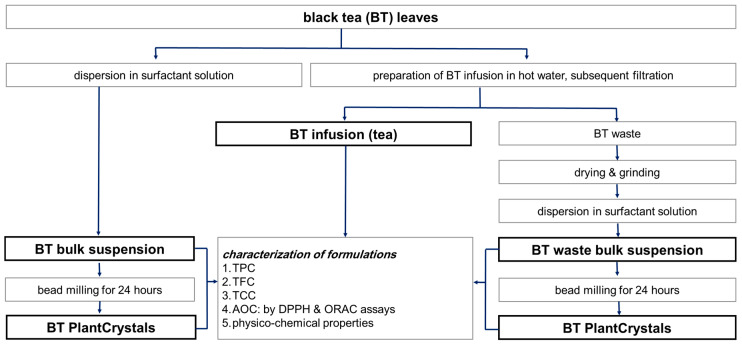
Scheme of the study showing the production and characterization of PlantCrystals from black tea (BT) and its waste. TPC: total polyphenol content, TFC: total flavonoid content, TCC: total carotenoid content and AOC: antioxidant capacity by using DPPH (1,1-diphenyl-2-picrylhydrazyl) and ORAC (oxygen radical absorbance capacity) assays. Physico-chemical properties include: particle size, morphology and zeta-potential.

The aim of this study is to systematically analyze the properties of the BT waste and to introduce its antioxidative molecules using the new green PlantCrystals extraction technology. To improve the AOC of BT waste and extract the insoluble molecules with antioxidative properties without using organic solvents, BT waste was milled to sizes well below the plant cell size. Since it is demonstrated that this PlantCrystal technology is suitable to improve the extraction efficacy of active compounds upon plant nanomilling procedure, total polyphenol content (TPC), total flavonoid content (TFC), total carotenoid content (TCC) must be determined [5]. Furthermore, fresh BT leaves were nano milled to compare its PlantCrystals antioxidant capacity to those of waste PlantCrystals. Both PlantCrystals formulations and their corresponding bulk-suspensions were also compared with BT infusion, which served as a control extract (Figure 2).

In brief, the study was performed in three parts. In the first part, BT infusion was prepared, its TPC, TFC, TCC and antioxidant capacity (AOC) were determined. AOC was determined by electron transfer (ET) and hydrogen atom transfer (HAT) mechanisms using DPPH^●^ (1,1-diphenyl-2-picrylhydrazyl) and ORAC (oxygen radical absorbance capacity) assays, respectively. The BT infusion was used as a control extract in this study. The resulted waste was collected and dried at room temperature. In the second part, bulk suspensions of the BT leaves and dried waste were produced by suspending the dried plant powder in a surfactant solution. The surfactant solution was used to physically stabilize the produced plant particles and prevent their agglomeration. These bulk suspensions were also characterized regarding size, TPC, TFC, TCC and AOC. In the third part of the study, PlantCrystals technology was utilized to activate the plant antioxidants. To achieve this, small-scale bead milling was performed to break the plant cells and produce PlantCrystals. All produced PlantCrystal formulations were characterized and compared with the bulk materials and tea infusion regarding physico-chemical properties, TPC, TFC, TCC and AOC using the previously mentioned assays. PlantCrystals physico-chemical properties were analyzed using light microscopy, laser diffraction, dynamic light scattering and zeta potential measurements.

## 2. Results

### 2.1. Black Tea (BT) Infusion

Classical aqueous BT extract (infusion) with 1% (*w*/*w*) of the dried and unprocessed plant material in hot purified water (as extraction medium) was used as a control extract. This control extract was characterized regarding polyphenol, flavonoid, carotenoid contents and antioxidant capacity (AOC) and the results are shown in Table 1. TPC of the BT infusion was used to determine the amount of hot water-soluble polyphenols, e.g., gallic acid and the value indicated a high amount of extracted polyphenols from the BT infusion. In addition, total flavonoid content (TFC) and total carotenoid content (TCC) were detected with low values of 9 mg QE/g and 2 mg β-carotene/g, respectively (Table 1). These low TFC and TCC values can be explained by hydrophobic antioxidants from plants, which are usually poorly water-soluble flavonoids and carotenoids. In other words, in the BT infusion only the water-soluble flavonoids, e.g., theaflavins and thearubigins are presented. These compounds contribute to the BT color, brightness and taste as well as its high AOC. The low TCC value of BT infusion was expected, to analyze the extraction efficacy of the PlantCrystal technology it was necessary to measure it nonetheless.

In the next step, AOC of the BT infusion was determined by using DPPH^●^ (1,1-diphenyl-2-picrylhydrazyl) and ORAC assays. AOC using DPPH assay was expressed as IC 50 values, which represent the number of active constituents needed to scavenge 50% of a given amount of free radicals, i.e., low IC 50 values represent a high AOC. This IC 50 value of the BT infusion was similar to the used standard (ascorbic acid (0.008 mg/ml)) as there is no significant difference between their IC 50 values. ORAC assay results also confirmed the high AOC of the tea infusion and showed the highest ORAC value of 55 µmol TE/µg (Table 1). The high AOC values of the tea infusion can be explained by the extraction of the hot water-soluble antioxidants.

### 2.2. Bulk-Suspensions from Black Tea (BT) Leaves and Waste

The bulk-suspensions were produced by mixing the plant powder with surfactant solution (Plantacare2000^®^ 1% *w/w*) to obtain a final concentration of 1% (*w/w*) plant suspension.

**Figure 3:**
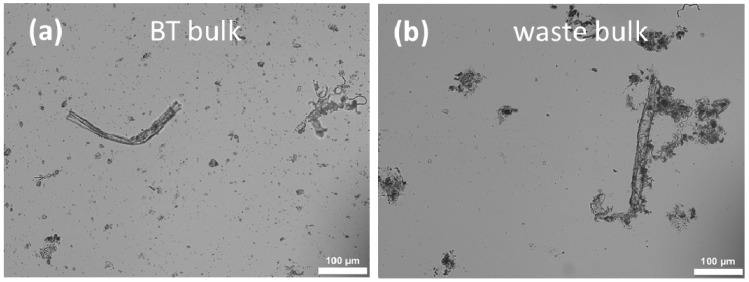
Light microscopic images of the produced bulk-suspensions: black tea (BT) leaves **(a)** and their waste **(b)** (200-fold magnification).

**Figure 4:**
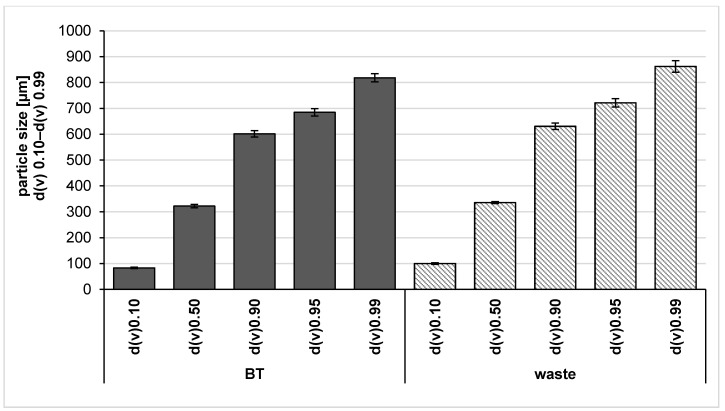
Laser diffraction data showed particle size of the bulk-suspensions obtained from black tea (BT) leaves and their waste.

The obtained BT bulk suspension and waste bulk suspension were characterized regarding size using light microscopy (LM) and laser diffraction (LD). LM images showed plant parts with irregular shapes randomly distributed in the bulk materials of black tea and its waste (Figure 3). LD analysis was used to compare the size distributions of the particles visualized by using microscopic images. The size profile is illustrated in Figure 4 and showed similar results for the two bulk suspensions as they possessed a size of about 800 µm (d(v)0.99 value), meaning that 99% of the measured particles were below 800 µm.

**Figure 5:**
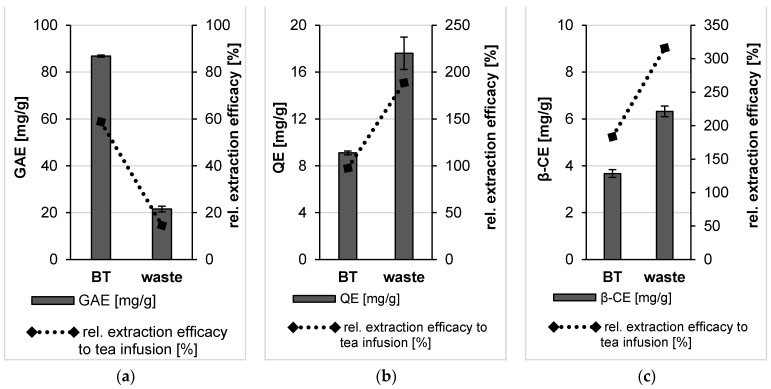
Determination of total polyphenol (**a**), flavonoid (**b**) and carotenoid contents (**c**) of the bulk suspensions from BT leaves and their waste. The polyphenol content is expressed in gallic acid equivalents (GAE), the flavonoid content is expressed in quercetin equivalents (QE) and the carotenoid content is expressed in β-carotene equivalents (β-CE).

The determination of total polyphenol content revealed a high TPC value for the bulk material obtained from BT leaves and about 4-fold lower TPC value of the bulk suspension obtained from the waste (Figure 5). In contrast to this, the TFC value of the waste bulk suspension was doubled in comparison to the bulk suspension of the BT leaves, this difference was not significant. However, the TCC value of the bulk waste was 2-fold higher than the BT leaves bulk suspension. This difference was significant (Figure 5).

**Figure 6:**
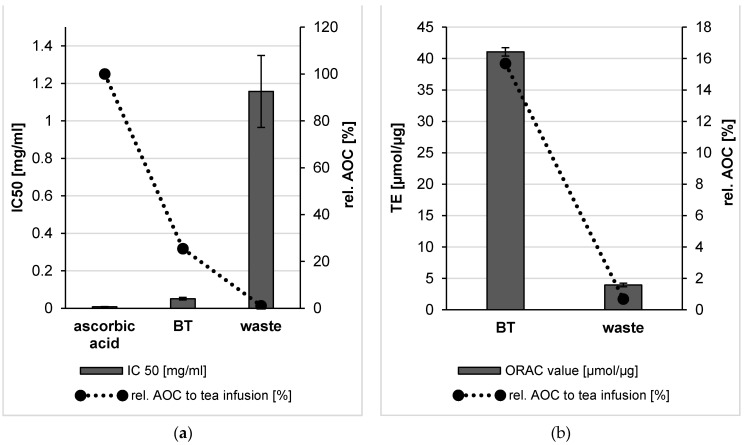
Antioxidant capacity of BT leaves and their waste bulk suspensions using DPPH and ORAC assays. The results of the DPPH assay were expressed as IC 50 and compared to the used standard in this study (ascorbic acid) (**a**). ORAC values were expressed as µmol Trolox equivalent per µg of the sample analyzed (**b**).

According to the DPPH assay, the BT bulk suspension showed a low IC 50 value which proves its high AOC. However, the IC 50 value of the bulk suspension from the waste indicated a very weak AOC with a decrease more than 20 times in comparison to the BT leaves bulk materials (Figure 6). Both bulk suspensions produced in this study showed significantly lower AOCs (*p* < 0.05) when compared to the used standard (ascorbic acid).

ORAC method is characterized by capturing endogenous radicals that act on oxidized targets and, thus, it is a meaningful tool to express the antioxidant capacity (AOC) of a formulation. The ORAC value of the bulk material of black tea was found to be 41 µmol Trolox equivalent per µg (µmol TE/µg), which proved the high antioxidant activity of black tea. In contrast, the low ORAC value of the waste bulk materials indicates the occurred washing out of the soluble phytochemicals upon tea preparation (about 11-fold less than the ORAC value of the BT bulk materials) (Figure 6). The explanation for the high AOC of the tea infusion lies in the soluble compounds. These high amounts of water-soluble antioxidants were released during tea preparation and without the need for diminution of the tea leaves by BM. Literature also proves that black tea contains extremely high amounts of water-soluble antioxidants [20].

### 2.3. PlantCrystals from Black Tea (BT) Leaves and Waste

The PlantCrystal technology is a simple method to produce ecofriendly and sustainable plant extracts [5]. The process does not require organic solvents and can be scaled up easily. It is thus industrially feasible and can be applied to the extraction of many plant molecules. This PlantCrystals extract can be obtained by nanomilling and is especially suitable for the extraction of lipophilic and poorly water-soluble compounds. A small-scale bead milling technique was successfully applied in this study to obtain BT waste PlantCrystals which were compared to BT leaves PlantCrystals.

**Figure 7:**
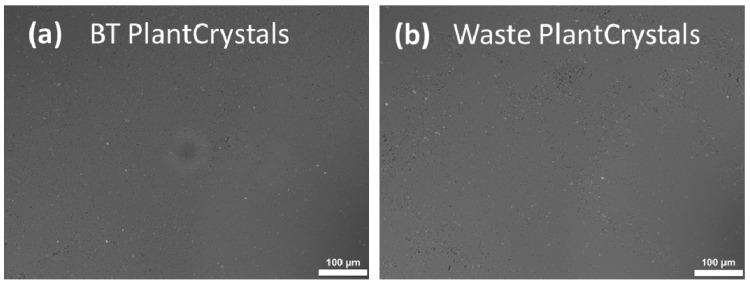
Light microscopic images of the obtained PlantCrystals: black tea (BT) leaves **(a)** and their waste **(b)** (200-fold magnification).

To view the produced PlantCrystals, they were observed using a light microscope after nanonization. Light microscopy of both PlantCrystals produced from BT and its waste by bead-milling confirmed that they did not reveal big, visible particles, which indicates the formation of finer PlantCrystals (Figure 7).

**Figure 8:**
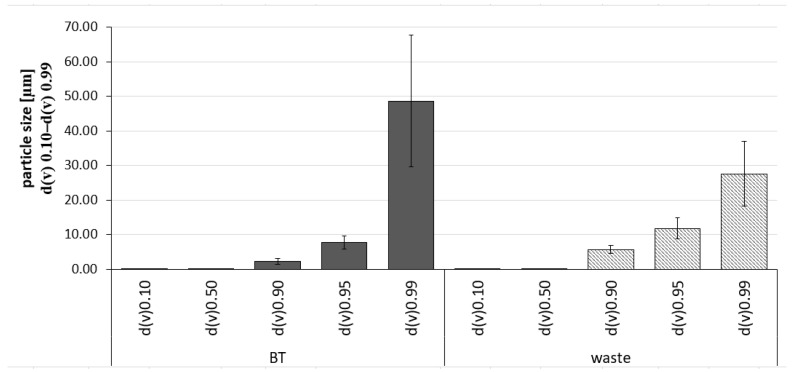
Laser diffraction data showing particle size of the PlantCrystals obtained from black tea (BT) leaves and their waste.

Further characterization using the LD revealed some larger sized particles with sizes of about 49 μm (d(v)0.99 value) (Figure 8), which were not minimized during the production process. However, 95% of the volume of the particles (d(v)0.95 value) possessed sizes < 10 µm, which is the approximate size of a plant cell. In other words, the number of these large particles seems to be very small, because light microscopy did not reveal such big particles or agglomerates for the PlantCrystals produced. Light microscopic images showed a great decrease in particle size in the viewed PlantCrystals compared to the bulk materials (Figure 7). In contrast, LD analysis of the BT waste revealed that the PlantCrystals also contained some large particles with sizes of about 28 μm (d(v)0.99 value) (Figure 8). Meanwhile, 90% of the volume of the particles possessed sizes below 10 µm. Hence, almost all cells of black tea and its wastes were nano-milled by the small-scale bead milling process to sizes well below the approximate size of the plant cell.

In brief, LD data of both PlantCrystals from BT and its waste showed that 90% of the volume of the particles possessed sizes well below 10 μm, but only a few particles possessed sizes in micron level (Figure 8).

Dynamic light scattering (DLS) was also used to characterize the PlantCrystals. This technique was used to measure nanoscaled particles that were not revealed in the LD measurements. Black tea PlantCrystals possessed a particle size of about 340 nm and a polydispersity index (PdI) value of about 0.4 (±0.06) according to the DLS results (Table 2). However, nanonization process applied to the waste of the black tea bulk material reduced the sizes of the bulk materials to smaller and more homogenous PlantCrystals with a particle size of about 280 nm and PdI value of about 0.4 (±0.02) as shown in the DLS data (Table 2).

Zeta-potential (ZP) is considered a vital parameter in nano-formulations to predict their physical stability. Therefore, ZP was measured in water with a conductivity of 50 μS/cm. The measurement was carried out to detect the charges on the PlantCrystals surface. A high value indicates good physical stability of the produced PlantCrystals. The obtained ZP values are −24 (±0.1) and −27 (±0.8) mV in the final formulation of the black tea and its waste, respectively, indicating that the produced PlantCrystals in this study are physically stable.

**Figure 9:**
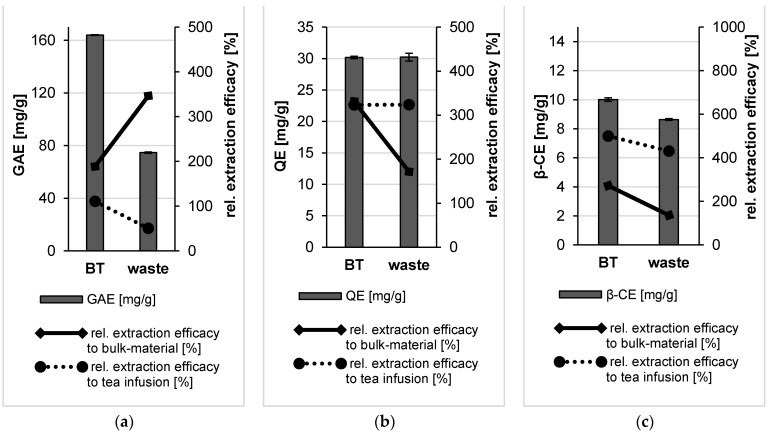
Determination of total polyphenol (**a**), flavonoid (**b**) and carotenoid contents (**c**) of the PlantCrystals from BT leaves and their waste. The polyphenol content is expressed in gallic acid equivalents (GAE), the flavonoid content is expressed in quercetin equivalents (QE) and the carotenoid content is expressed in β-carotene equivalents (β-CE).

The determination of total polyphenol content demonstrated the efficacy of the plant milling procedure applied on BT and its waste. A pronounced increase in TPC value was observed upon bead milling of BT and its waste as the content of those active constituents in the PlantCrystals formulations was higher compared to the corresponding bulk suspensions (Figure 9). Thus, the application of small-scale BM process on black tea increased the available polyphenols in black tea PlantCrystals showing the highest TPC value among the analyzed formulations (Figure 9) but this increase was not significant if compared with the BT bulk suspension. However, a pronounced increase in TPC (more than 3-fold) was observed after the nanonization of the dried waste (Figure 9). Thus, BM could increase the extraction efficacy of these molecules of BT waste in a significant way (*p* < 0.05) vs. the released molecules from the bulk suspension. 

Furthermore, total flavonoid content (TFC) showed a 3-fold and 1.7-fold increase in the TFC value for the PlantCrystals from black tea and its waste compared to the corresponding unprocessed bulk suspension, respectively (Figure 9). BT PlantCrystals showed a value of about 30 mg QE/g, which was much higher than bulk materials of BT and tea infusion presenting similar values of about 9 mg QE/g (Figure 9). Despite the increase in TFC value upon nanomilling processes it was not significant in comparison to the corresponding bulk materials.

The determination of the carotenoid content demonstrated the efficacy of the plant milling procedure (Figure 9). After BM, the carotenoid content was increased about 3-fold compared to the bulk material of black tea and about 36% for the black tea waste compared to the bulk waste. The carotenoid content after nanomilling was significantly increased. The data, therefore, support the theory that the PlantCrystal technology is especially suitable for the improved extraction of lipophilic compounds of plant materials.

**Figure 10:**
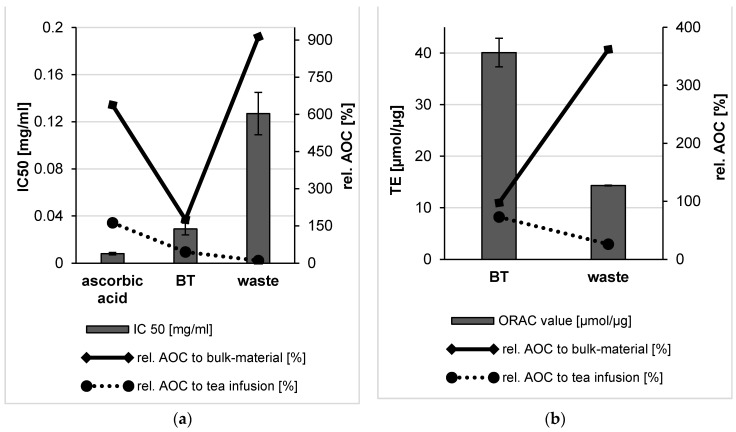
Antioxidant capacity of BT leaves and their waste PlantCrystals using DPPH and ORAC assays. The results of the DPPH assay were expressed as IC 50 and compared to the used standard in this study (ascorbic acid) (**a**). ORAC values were expressed as µmol Trolox equivalent per µg of the sample analyzed (**b**).

A DPPH assay was performed to determine the free radical scavenging capacity of the produced formulation and the data showed that nanonization almost doubled the AOC of BT leaves, when compared to the bulk material (Figure 10). However, the AOC was about 4 times higher when the leaves were brewed to yield a classic black tea infusion. In contrast, PlantCrystals obtained from the waste showed a well improved AOC in comparison to the bulk waste (*p* < 0.001), hence the AOC increased with decreasing the size as the antioxidative potential increased about nine-fold upon nanonization (Figure 10). The IC 50 of the waste PlantCrystals was as good as the tea infusion and the used standard (ascorbic acid) as there is no significant difference between their IC 50 values. Despite the higher AOC of the BT PlantCrystals in comparison to the waste PlantCrystals, a significant difference between the bulk and nanosized waste was observed. However, no significant difference was observed between the bulk and nanosized fresh BT leaves. In brief, according to the DPPH assay, the black tea infusion had the best AOC followed by the obtained PlantCrystals from the black tea leaves and then their waste with IC 50 values of 0.013, 0.029 and 0.127 mg/ml, respectively.

In order to provide reliable and more detailed information about the AOC of the produced plant extracts by using the PlantCrystals technique, ORAC assay was chosen with a different principle, i.e., hydrogen atom transfer (HAT). The ORAC assay is considered to be most suitable to assess hydrophilic and lipophilic antioxidants at the same time. However, the ORAC value of BT leaves was slightly decreased (40 µmol TE/µg) after nanonization (Figure 10). In contrast, the ORAC value of the obtained waste PlantCrystals significantly increased about 4 times compared to the bulk waste. Thus, the ORAC value of the waste increased upon the nanonization process, leading to a pronounced AOC. The analyzed PlantCrystals from the tea and its waste showed a significant difference (*p* < 0.05) compared to the tea infusion, which showed the best ORAC value and therefore the best AOC (55 µmol TE/µg). 

## 3. Discussion

Black tea is the most popular prepared drink worldwide. Therefore, a huge amount of black tea wastes containing a fortune of antioxidant active molecules occurs as a result. The applicability of the PlantCrystal technology to recycle this plant waste as antioxidative formulations was proven in this study. 

The data obtained demonstrated that milling of the black tea leaves and their waste to sizes < 10 µm was successfully achieved by using small-scale bead milling (BM). The process is considered a green method as no organic solvents are required. It leads to the effective destruction of all plant cells and cell organelles and thus to the release of more physiologically active molecules with antioxidative properties. 

The antioxidants released after nanomilling are more lipophilic, due to the destruction of lipophilic cell compartments that host these compounds [5]. Consequently, smaller sizes of the PlantCrystals can be expected to cause a higher release of lipophilic antioxidants, and thus, led to improved AOCs. 

Oxidation can happen during the production process for both hydrophilic and lipophilic molecules, but hydrophilic antioxidants are more prone to the oxidation process. In the bulk suspensions, the particles are composed of several cells, so that the lipophilic compounds inside the cells (before the nanonization process) are protected from oxidation. In the next step (after nanomilling), the oxidation process can also occur on the released lipophilic antioxidants. However, the surfactant used in the production of PlantCrystals solubilizes the lipophilic compounds directly upon their release from the cell compartments into the hydrophobic core of the micelles and thus protects them from oxidation [5]. Thus, nanomilling of plants is considered more suitable for the lipophilic molecules.

On one hand, size characterization methods used in this study proved the presence of particles well below 10 µm (the approximate plant cell size). This led to the release of the molecules associated with antioxidative properties from black tea and its waste. On the other hand, zeta-potential can be used to determine the physical stability of the PlantCrystals. The measurement was carried out to detect the charge on the PlantCrystals surface. A high value indicates good stability of the suspensions [21]. The zeta-potential values of the produced PlantCrystals indicated decent stability showing values between −23 mV and −27 mV in the final formulation. This stability could be due to the steric stabilization effect of the nonionic surfactant. 

The higher polyphenol, flavonoid and carotenoid contents of those active constituents in the PlantCrystals formulation might be attributed to the destruction of plant cells, which causes an exhausting release of all active constituents. In contrast to tea infusion, where mainly hot water-soluble phytochemicals are released, the poorly water-soluble active constituents in the PlantCrystals are also released. Interestingly, tea infusion showed a higher TPC value in comparison to the bulk waste but PlantCrystals from the waste showed a significant increase (more than 3-fold) after nanonization. This indicates the occurred washing out of hot water-soluble phytochemicals upon tea infusion preparation and releasing of the remaining insoluble ones upon nanonization. Nonetheless, it has to be mentioned that the Folin–Ciocalteu method overestimates the TPC value due to the lack of selectivity of the Folin–Ciocalteu reagent, which reacts not only with phenols but also with other reducing compounds such as carotenoids, amino acids, sugars and ascorbic acid [22]. The increased TPC values after nanomilling thus indicate an increased amount of reducing agents in the formulation and thus higher AOC. Without further detailed studies, it cannot be concluded that only polyphenols account for this increase.

The higher TFC value of the bulk obtained from the waste in comparison to the tea infusion can be explained by the improved effect of the surfactant solution to extract these flavonoids, i.e., the increased solubility of the waste flavonoids in the surfactant solution. However, PlantCrystals from both, the tea leaves and their waste, showed a pronounced increase in the TFC value indicating the release of the insoluble flavonoids after breaking the plant cells by using small-scale bead milling.

Based on the previous findings, the determination of the carotenoid content demonstrated the efficacy of the plant milling procedure because of their poor water-solubility. In this study, it was demonstrated that even if only low amounts of these compounds are available, a pronounced release after the nanomilling process (in comparison to the bulk materials and the aqueous extract) proofs the efficacy of the plant milling technology. When compared to the tea infusion, all suspensions produced in this study showed higher carotenoid content. This can be explained by the rupture of the plant cells which causes the increased releases and—in addition—the effect of the surfactant which solubilized some of the lipophilic phytochemicals in the bulk suspensions. The data, therefore, substantiate the theory that the PlantCrystal technology is especially suitable for improved extraction of lipophilic compounds.

Nevertheless, this study demonstrated that the produced PlantCrystals possess the highest polyphenol, flavonoid and carotenoid contents, i.e., the available content was higher due to the release that occurred upon small-scale BM process. This confirmed our hypothesis and agreed with our previous findings [5], that higher amounts of poorly water-soluble plant active constituents were released from the PlantCrystals when compared to bulk material and the used control extract (tea infusion).

Antioxidant capacity must be evaluated by a number of methods to consider the different modes of action of a particular antioxidant [23]. Therefore, in this study, DPPH and ORAC assays were used, which are based on electron transfer (ET) and hydrogen atom transfer (HAT) mechanisms, respectively. The results of both assays proved that the BT bulk-suspension possesses a high AOC, but show no significant difference after nanonization. In contrast, the bulk of black tea waste showed weak antioxidant activity, which was significantly improved by using the natural, ecofriendly and cost-effective PlantCrystal- technology, i.e., the obtained “Nano-Wastes” (PlantCrystals obtained from organic waste) demonstrated a better antioxidant activity compared to its bulk and this agreed with a study performed by Griffin et al. in which the authors could prove that nanonization can unlock the antioxidant potential remaining in the plants waste [3]. This higher AOC of the produced BT waste PlantCrystals in this study is related to the higher extraction efficacy shown in the higher TPC, TFC and TCC values after nanomilling process (Table 3). However, according to the DPPH assay, the tea infusion (control extract) had the best AOC followed by the obtained PlantCrystals. This can be due to the higher extraction efficacy of the soluble black tea polyphenolic catechins in boiling water, since BT contains extremely high amounts of water-soluble compounds [20,24]. Lipophilic compounds are poorly soluble in water. Therefore, it is highly likely that the surfactant used in the formulation solubilizes these compounds immediately upon their release from the plant cells into the aqueous phase. This means that the lipophilic antioxidants are localized in the hydrophobic core of the micelles and thus are more protected from oxygen than the hydrophilic antioxidants. Thus, the AOC of the final product represents the sum of all antioxidants released during the comminution reduced by the number of antioxidants that were degraded during the nanomilling process. In other words, the final AOC is the total of the native constituents and the ones produced during the milling process.

The used surfactant might have an impact on the AOC of the produced formulation. However, it has recently been reported by Stahr et al. that this impact is not significant [25].

Nevertheless, recycling plant waste, e.g., black tea waste, was achieved by using PlantCrystal technology to improve its AOC and render it into plant medicinal products and/or cosmetics with higher amounts of plant active molecules and without the use of organic solvents. This makes PlantCrystal technology highly suitable for the production of sustainable medicinal plant products.

Hence, the antioxidant activity of phytochemicals is not limited to the free radical scavenging ability and/or oxygen radical absorbance. It also involves the inhibition of the oxidation of lipoproteins in cell membranes in addition to heavy metals chelating activity, which can be tested using lipid peroxidation inhibition capacity assay and the 2,2’-azino-bis(3-ethylbenzothiazoline-6-sulphonic acid) (ABTS^●^) radical assay, respectively. It is recommended for the upcoming studies to perform more antioxidant assays in order to detect the total AOC of PlantCrystals from black tea and its waste. In addition, it has to be stated that all particles scatter light, meaning that scattered light from the particles can interfere with the UV/vis measurements to some extent. At present, the issue is addressed by using samples with extremely low particle concentration and in the future improved methods for such kind of formulations (PlantCrystals) should be developed. Nonetheless, the present methods can already provide detailed and discriminative information on the activity of the produced formulations.

In spite of this, it is worth saying that the obtained results are useful to further identify the specific polyphenolic compounds responsible for the antioxidant activities and to study their structure–function interactions, i.e., to specify the released compounds upon nanonization and enable the development of pharmaceutical and cosmeceutical products.

## 4. Materials and Methods 

### 4.1. Production of Black Tea Infusion and the PlantCrystals

Black tea (Royal Birdsong Tea Caddy) was bought from Buckingham Palace shop in London, United Kingdom in December 2017. The teabags were emptied and the dry coarse leaves (with size ≥1 mm) were pre-grinded using mortar and pestle and hand blenders (Elta Lizenz GmbH, Oststeinbek, Germany and AR1105 Moulinex, Grenoble, France). A solution of Plantacare2000 1% *w*/*w* (Sigma-Aldrich, Darmstadt, Germany) was used as a suspending agent to stabilize the produced plant particles and prevent any possible agglomeration. Plantacare2000 is a natural obtained non-ionic surfactant composed of decyl glucoside [26,27]. To prepare the Plantacare2000 1% *w*/*w* solution, purified water was used as a dispersion medium and was obtained from a PURELAB Flex 2 (ELGA LabWater, High Wycombe, UK).

Black tea infusion was used as a control extract. Therefore, 1 g tea leaves were boiled in 100 g water (without surfactant) for five minutes [28]. After this, the obtained aqueous extract was cooled at room temperature and then the mixture was filtered through a metal sieve to collect the coarse BT material. Subsequently, the supernatant was filtered through filter paper with a pore size of 16 µm to collect also smaller sized plant material. The obtained BT aqueous extract was immediately used for further analysis. The obtained residue was collected, dried and stored at room temperature for less than 24 hours to be used in the following part of the experiment.

Black tea leaves and dried waste were subjected to dry milling using mortar and pestle and hand blenders in situ. In the next step, 1 g of the dried and grinded black tea leaves was suspended in 100 g Plantacare2000 surfactant solution and stirred for about 15 minutes using a magnetic stirrer to obtain the macro bulk-suspension (BT bulk-material). In addition, 1 g of the collected, dried and ground waste was treated likewise. The PlantCrystals were produced using small-scale bead milling in a ratio of 60:40 of plant bulk suspensions to the used Yttrium stabilized zirconium oxide beads (Ø 1 mm, SiLibeads^®^, Sigmund Lindner GmbH, Warmensteinach, Germany). The method involves the production of PlantCrystals through pearl mills. The produced bulk-suspensions were nano-milled in a milling chamber composed of small vials (size 12 × 35 mm) containing Yttrium stabilized zirconium oxide beads as milling medium and magnetic stirring rods (size 6 × 10 mm) as a milling shaft. The milling media and pearls were then rotated using a magnetic stirrer (IKA-Combimag RCT, IKA, Staufen, Germany) at 1500 rpm for 24 h at room temperature [5,29].

### 4.2. PlantCrystal Size Analysis

#### 4.2.1. Light Microscopy (LM)

The particles were assessed regarding particle shape by using light microscopy (Olympus BX53 microscope, Olympus Corporation, Tokyo, Japan) equipped with a SC50 CMOS color camera (Olympus soft imaging solutions GmbH, Münster, Germany). 

#### 4.2.2. Laser Diffraction (LD)

All the produced samples were analyzed using laser diffractometry (Mastersizer 3000, Malvern-Panalytical, Kassel, Germany). The measurements were conducted for six-times and the average values were represented as the mean ± standard deviation (SD). The used dispersion medium was the Hydro S sample dispersion unit. All parameters were analyzed using Mie theory [3,7]. The used real refractive index (RI) was 1.592 and the imaginary refractive index (IRI) was 0.01. The data of laser diffraction are usually shown as volume size distribution and particles up to 2000 µm can be measured using this technique [30].

#### 4.2.3. Dynamic Light Scattering (DLS)

The DLS analysis was carried out using the Zetasizer Nano ZS (Malvern-Panalytical, Kassel, Germany). Measuring conditions were adjusted at 20 °C for the produced PlantCrystals. The data were analyzed with the general-purpose mode built in the software of the instrument [3,7]. DLS yields a light intensity weighted mean diameter (z-average) as a measure for the size of the PlantCrystals and the polydispersity index (PdI) as a measure for the width of the size distribution. DLS can detect particle sizes between 3 nm to 3 µm. The analysis was performed in triplicate and the results are expressed as means ± standard deviation.

#### 4.2.4. Zeta-Potential (ZP) 

The zeta-potential represents the electric potential at the slipping plane of the electrical double layer. It is considered a vital parameter in nano-formulations to predict their physical stability. The ZP was measured at 20 ℃ using Zetasizer Nano ZS (Malvern-Panalytical, Kassel, Germany). The measurement was performed in conductivity adjusted purified water (50 μS/cm) [21]. A laser-Doppler-anemometry (LDA) was used to determine the electrophoretic mobility, which was then converted into the ZP by using the Helmholtz–Smoluchowski equation according to the Malvern Zetasizer software. Measurements were performed in triplicate.

### 4.3. Total Content and Antioxidant Capacity (AOC) Tests

#### 4.3.1. Total Polyphenol Content (TPC)

The Folin–Ciocalteu method was used to analyze the total polyphenolic content for the BT infusion and the other formulations produced in this study [5,31,32]. The assay was performed in a 96-well plate. The reaction mixture contained purified water, suspensions, BT infusion or standard, 20% sodium carbonate and 2N Folin–Ciocalteu reagent (Merck KGaA, Darmstadt, Germany) at a ratio of 7.5:1:1:0.5. The reaction mixture was then incubated in the dark for 60 minutes at room temperature. After this, the absorbance was recorded at 765 nm using a multi-plate UV/VIS spectrophotometer (Multiskan GO, Thermo Scientific, Dreieich, Germany). The TPC values were calculated as mg gallic acid (Thermo Scientific, Waltham, MA, USA) equivalents per gram of sample (mg GAE/g). Rel. extraction efficacy was calculated in comparison to the used control extract and/or the unprocessed bulk materials. Experiments were performed in triplicate.

#### 4.3.2. Total Flavonoid Content (TFC)

The TFC was determined using a method based on the interaction of flavonoids with AlCl_3_ that leads to the formation of a complex that can be determined via UV/Vis spectroscopy using a multi-plate UV/VIS spectrophotometer at 420 nm (Multiskan GO, Thermo Scientific, Dreieich, Germany) [5,33]. A calibration curve with quercetin (10–100 µg/mL, Biomol GmbH, Hamburg, Germany) was used. The samples were diluted with purified water prior to the experiment and the results were expressed as mg quercetin equivalents per gram of sample (mg QE/g) based on the used calibration curve. Rel. extraction efficacy was calculated in comparison to the used control extract and/or the unprocessed bulk materials. Experiments were performed in triplicate.

#### 4.3.3. Total Carotenoid Content (TCC)

TCC was determined by the method of Rodriguez-Amaya et al. [5,34]. In brief, the absorbance of each formulation and tea infusion was measured at 450 nm (Multiskan GO, Thermo Scientific, Dreieich, Germany) after dilution with purified water and calculations were performed with respect to a standard curve of the beta-carotene (TCI Deutschland GmbH, Eschborn, Germany). Results are expressed as mg beta-carotene equivalent per gram of sample (mg β-CE/g). Experiments were performed in triplicate. Rel. extraction efficacy was calculated in comparison to the used control extract and/or the unprocessed bulk materials.

#### 4.3.4. DPPH^●^ (1,1-diphenyl-2- picrylhydrazyl) Assay

The antioxidant capacity of the produced PlantCrystals of the black tea, its waste and the tea infusion used in this study was investigated using the DPPH^●^ (Sigma–Aldrich Chemie GmbH, Steinheim am Albuch, Germany) assay according to the method proposed by Sharma and Bhat [3,5,6,31,35] and compared to AOC of the bulk materials, where ascorbic acid (Sigma Chemical Co., Louis, MO, USA) was used as standard (lowest IC 50). The test was performed on a 96-well plate. Initially, a 0.2 mM DPPH solution was prepared in methanol. Afterward, a series of dilutions of each sample (100, 50, 25, 12.5, 6.25, 3.125, 1.562 μg/ml) were prepared using purified water and finally 100 µl of DPPH solution was added. Methanol in addition to purified water was used as blanks. That was followed by incubating the plates in dark for 30 minutes to allow the reaction to occur. Subsequently, the absorbance was measured by a multi-plate UV/VIS spectrophotometer at 517 nm (Multiskan GO, Thermo Scientific, Dreieich, Germany). Results are expressed as IC 50 value (mg/ml) and as rel. AOC in comparison to the used control extract and/or the unprocessed bulk materials. Experiments were performed in triplicate and then the percentage of radical scavenging activity (RSA) values were calculated from the following Equation (1):

Radical scavenging activity (%) = (Abs. _DPPH_ − Abs. _sample_\Abs. _DPPH_) × 100 (1)

Abs. _DPPH_ is the DPPH absorbance and Abs. _sample_ is the sample absorbance. The sample concentration and their corresponding %RSA were plotted in a graph to obtain the corresponding IC 50-values, i.e. the amount of formulation needed to scavenge 50% of a given amount the free radicals.

#### 4.3.5. ORAC (Oxygen Radical Absorbance Capacity) Assay

ORAC assay was performed following the previously described procedure [5,36]. In brief, the assay was performed in black opaque 96-well plates. AAPH (2,2’-azobis(2-methylpropionamidine) dihydrochloride, Acros Organics, Geel, Belgium) was used as a peroxyl radical generator, fluorescein (Alfa Aesar, ThermoFisher GmbH, Kandel, Germany) was used as fluorescent and Trolox (6-Hydroxy-2,5,7,8-tetramethylchroman-2- carboxylic acid, Santa Cruz Biotechnology Inc., Dallas, TX, USA) was used as a standard. Fluorescein intensity was measured (FluoStar^®^ Optima plate, BMG Labtech, Offenburg, Germany) every minute for 80 minutes in total at excitation and emission wavelengths of 485 and 520 nm, respectively. A Trolox standard curve was prepared in the range (22.5–100) μM and ORAC values of the tested formulations were calculated and expressed as µmol Trolox equivalents per µg of the sample. In addition, results are expressed as rel. AOC in comparison to the used control extract and/or the unprocessed bulk materials. Experiments were performed in triplicate.

#### 4.3.6. Statistical Analysis

All results were expressed as mean ± SD. All statistical analyses were performed using GraphPad Prism 5 (GraphPad Software Inc., San Diego, CA, USA). Analysis of variance and Tukey’s multiple comparison test were performed to evaluate significant (*p* < 0.01) differences between bulk-materials, PlantCrystals and tea infusion.

## 5. Conclusions

PlantCrystals from bulk materials of BT waste were successfully produced by small-scale bead milling technique. The bulk-suspension of BT waste possesses weak antioxidant activity, which was significantly improved by the nanonization process. Hence, PlantCrystal technology is an efficient way to be applied on BT waste to increase the extraction efficacy of its antioxidative molecules and thus to be processed and recycled in pharmaceutical compounds and cosmetics. Therefore, it can be concluded that BT wastes (available in huge amounts and considered as a rich and sustainable source of phytochemicals) nanonization can increase its antioxidative properties.

Based on the results of this study, it can be concluded: nanomilling is also applicable on plants wastes, e.g., black tea waste, and leads to higher amounts of plant active constituents release (in comparison with the unprocessed corresponding bulk materials) without the use of organic solvents. Thus, making the PlantCrystal technology a highly suitable technology for the production of sustainable plant products for use in food, cosmetic and medicinal products. 

The PlantCrystal technology represents a natural, ecofriendly and cost-effective alternative source of antioxidant compounds even from plant wastes. Therefore, we can conclude that PlantCrystals from black tea waste were produced with improved antioxidant capacity.

## Figures and Tables

**Table 1:** Characterization overview of the used control extract BT infusion. Total polyphenol content (TPC) is expressed in gallic acid equivalents (GAE), total flavonoid content (TFC) is expressed in quercetin equivalents (QE) and total carotenoid content (TCC) is expressed in β-carotene equivalents (β-CE) of black tea (BT) infusion.

	TPC GAE [mg/g]	TFC QE [mg/g]	TCC β-CE [mg/g]	IC 50 [mg/mL]	ORAC-Value [µmol/µg]
Black Tea Infusion	148 ± 2	9 ± 0.2	2 ± 0.03	0.013 ± 0.005	55 ± 2

**Table 2:** Dynamic light scattering (DLS) data showing z-average and the polydispersity index (PdI) of the produced PlantCrystals of black tea (BT) leaves and their waste.

PlantCrystals	z-Average [nm]	PdI
BT	342 ± 7	0.4 ± 0.06
BT waste	279 ± 7	0.4 ± 0.02

**Table 3:** A comparison between content and antioxidant capacity (AOC) measurements of the produced PlantCrystals from black tea (BT) and its waste with their corresponding bulk suspensions. Total polyphenol content (TPC) is expressed in gallic acid equivalents (GAE), total flavonoid content (TFC) is expressed in quercetin equivalents (QE), total carotenoid content (TCC) is expressed in β-carotene equivalents (β-CE), IC 50 is giving the amount of active constituents needed to scavenge 50% of a given amount of free radicals and ORAC-values are expressed in Trolox equivalents (TE).

Sample	TPC GAE [mg/g]	TFC QE [mg/g]	TCC β-CE [mg/g]	IC 50 [mg/mL]	ORAC-Value TE [µmol/µg]
BT bulk-susp.	87 ± 0.4	9 ± 0.2	4 ± 0.2	0.051 ± 0.007	41 ± 1
BT PlantCrystals	164 ± 0.4	30 ± 0.2	10 ± 0.1	0.029 ± 0.005	40 ± 3
BT waste bulk-susp.	22 ± 1	18 ± 1	6 ± 0.2	1.157 ± 0.192	4 ± 0.3
BT waste PlantCrystals	75 ± 1	30 ± 1	9 ± 0.1	0.127 ± 0.018	14 ± 0.1

## Data Availability

Data is contained within the article.
